# RNA-sequencing reveals that STRN, ZNF484 and WNK1 add to the value of mitochondrial MT-COI and COX10 as markers of unstable coronary artery disease

**DOI:** 10.1371/journal.pone.0225621

**Published:** 2019-12-10

**Authors:** Paul Holvoet, Bernward Klocke, Maarten Vanhaverbeke, Roxane Menten, Peter Sinnaeve, Emma Raitoharju, Terho Lehtimäki, Niku Oksala, Christian Zinser, Stefan Janssens, Karin Sipido, Leo-Pekka Lyytikainen, Stefano Cagnin

**Affiliations:** 1 Department of Cardiovascular Sciences, KU Leuven, Leuven, Belgium; 2 Intrexon Bioinformatics Germany, Munich, Germany; 3 Department of Clinical Cardiology, UZ Leuven, Leuven, Belgium; 4 Department of Clinical Chemistry, Fimlab Laboratories, Tampere, Finland; 5 Finnish Cardiovascular Research Centre, Faculty of Medicine and Life Sciences University of Tampere, Tampere, Finland; 6 Division of Vascular Surgery, Department of Surgery, Tampere University Hospital, Tampere, Finland; 7 Department of Biology, CRIBI Biotechnology Centre, Padova, Italy; 8 CIR-Myo Myology Centre, University of Padova, Padova, Italy; University of Bologna, ITALY

## Abstract

Markers in monocytes, precursors of macrophages, which are related to CAD, are largely unknown. Therefore, we aimed to identify genes in monocytes predictive of a new ischemic event in patients with CAD and/or discriminate between stable CAD and acute coronary syndrome. We included 66 patients with stable CAD, of which 24 developed a new ischemic event, and 19 patients with ACS. Circulating CD14+ monocytes were isolated with magnetic beads. RNA sequencing analysis in monocytes of patients with (n = 13) versus without (n = 11) ischemic event at follow-up and in patients with ACS (n = 12) was validated with qPCR (n = 85). MT-COI, STRN and COX10 predicted new ischemic events in CAD patients (power for separation at 1% error rate of 0.97, 0.90 and 0.77 respectively). Low MT-COI and high STRN were also related to shorter time between blood sampling and event. COX10 and ZNF484 together with MT-COI, STRN and WNK1 separated ACS completely from stable CAD patients. RNA expressions in monocytes of MT-COI, COX10, STRN, WNK1 and ZNF484 were independent of cholesterol lowering and antiplatelet treatment. They were independent of troponin T, a marker of myocardial injury. But, COX10 and ZNF484 in human plaques correlated to plaque markers of M1 macrophage polarization, reflecting vascular injury. Expression of MT-COI, COX10, STRN and WNK1, but not that of ZNF484, PBMCs paired with that in monocytes. The prospective study of relation of MT-COI, COX10, STRN, WNK1 and ZNF484 with unstable CAD is warranted.

## Introduction

Several millions of patients in Western countries are hospitalized each year for chest pain. In approximately half of the cases, chest pain is of cardiac origin [1]. Among these patients approximately 50% exhibit underlying coronary artery disease (CAD) that eventually leads to an acute coronary syndrome (ACS). ACS encompasses the clinical spectrum ranging from unstable angina through acute myocardial infarction (AMI).

Since we aim to search biomarkers, a non-invasive approach by performing analyses in peripheral blood was considered to be more convenient and translatable to clinical practice. Moreover, since atherosclerosis is a systemic disease in which monocytes and derived macrophages play a crucial role, we believe that measuring monocyte behaviour in peripheral blood reflects the activity inside the coronary vessel wall. This view is also supported by previous findings where gene expression in peripheral whole blood samples appeared to mirror gene expression changes in the atherosclerotic vascular wall [2].

Previously, we measured members of the cytochrome oxidase (COX) IV complex, because it has been proposed that mitochondrial dysfunction resulting in mitochondrial oxidative stress contributes to development of age‐related metabolic changes and CAD [3, 4]. We demonstrated that low MT‐COI in monocytes of coronary artery disease patients identified a population at risk for new cardiovascular events [5]. However, we had selected cytochrome oxidase as a target a priori, and did not perform an unbiased analysis. Therefore, in this study we performed unbiased RNA sequence analysis followed by several modelling approaches to indentify the best prognostic markers predicting a future event in stable CAD patients and the best markers of ACS at time of blood sampling.

Thus, our first aim was to search for markers improving the prediction of a new ischemic event in stable CAD patients during a 5-year follow-up. Our second aim was to compare gene in stable CAD and ACS patients. We performed an exploratory RNA sequencing (RNA-Seq) analysis of RNA isolated from monocytes, precursors of macrophages, followed by selective quantitative validation of robustly differentially expressed genes with qPCR. We confirmed that MT-COI predicted a new event but that striatin (STRN) added to the power. In addition, we found that COX10 and zinc finger 484 were markers of ACS.

Then, we determined if those markers were related to cardiac troponin T in ACS patients, i.e. reflecting myocardial injury [6, 7]. To our surprise, the identified markers did not correlate with cardiac troponin, in this study we measured their expression in atherosclerotic plaques to determine their correlation with markers of vascular injury, in particular M1 macrophage markers. The current work identifies 2 novel markers in addition to members of the COX complex IV, which may improve discrimination between stable and unstable CAD patients.

## Materials and methods

### Patients and follow-up

All study subjects gave written informed consent. We did not include minors. The clinical study performed at University Hospital Leuven conforms to the principles outlined in the Declaration of Helsinki, and was approved by the Medical Ethics Committee of the University Hospital Leuven. We included 142 consecutive patients undergoing quantitative coronary angiography (QCA). In 66 patients, CAD was diagnosed as an epicardial coronary stenosis of at least 50% in at least 1 of the coronary arteries. This cohort was studied before, but in here we defined CAD as coronary stenosis of 50 instead of 30% [5]. These patients were all stable at time of blood sampling. Twenty-four (36.4%) of those 66 CAD patients experienced at least 1 new ischemic event during a 5-year follow-up: cardiovascular death (n = 2), ischemic stroke (n = 2), recurrent AMI (n = 1), and recurrent ischemia requiring recurrent ischemia requiring reintervention (n = 19, of which one patient had in-stent restenosis) [5]. Recurrent ischemia (n = 19) was defined as patients with signs of ischemia (ECG changes, angina pectoris, positive stress test), and significant new or progressive coronary lesions on angiography (n = 19, of which one patient had in-stent restenosis. As we hypothesized that monocyte behaviour is related to plaque stability, we included all major adverse cardiovascular events including stroke. Because ischemic stroke and acute myocardial infarction share similar biological processes, and they reflect a frequently used combined endpoint in clinical trials in both stable and unstable coronary artery disease stroke was also included as major cardiovascular event. The two cardiac deaths included one patient with refractory cardiogenic shock and electrical instability because of critical 3-vessel disease. The second patient developed ventricular fibrillation and recurrent MI because of severe in-stent restenosis and died shortly thereafter.

We also included 19 ACS patients, 5 with ST-segment elevation myocardial infarction (STEMI) and 14 with non-ST-elevation MI. Patients with a history of cancer, renal insufficiency, inflammatory disease, treatment with corticosteroids or immunosuppressive drugs, and significant valvular disease were excluded.

### Isolation of monocytes

For isolation of monocytes, blood was collected on BD Vacutainer^®^ CPT^™^ Mononuclear Cell Preparation Tube—Sodium Citrate (Becton Dickinson). CD14^+^ monocytes were isolated from the Histopaque-1077 leukocyte fraction using CD14 microbeads and LS column in a Midi-Magnetic Cell Isolation Separator (MACS, Miltenyi), as previously published [5]. Only monocytes wich were isolated within 2 hours of blood sampling were analyzed further. The purity of the isolated CD14+ monocytes was >95%, as previously published. The numbers of CD14+ monocytes isolated from 1 ml (approximately 2.5x10^5^) of blood were similar for stable CAD patients without and with a new ischemic event and ACS patients.

### RNA sequencing

RNA from monocytes was isolated at KU Leuven as described previously [5]. RNA sequencing (RNA-Seq) was performed by Biogazelle (Gent, Belgium) on a subset of patients which were randomly selected for RNAseq, as a discovery cohort. In the next step, validation with qPCR was performed in all patients. Libraries for mRNA sequencing were prepared using the TruSeq stranded mRNA sample prep kit (Illumina). In one run we analyzed 36 samples from three groups of patients. Group 1: stable CAD patients without new ischemic event (n = 11); group 2: stable CAD patients with new event (n = 13); group 3: ACS patients (n = 12) (see methods in S1 Appendix). We performed Mann Whitney test in search of transcripts distinguishing between stable CAD patients with and without a new ischemic event; thus ACS patients were not included. We also wanted to identify transcripts which separated ACS from the two groups of stable CAD patients. Therefore, we performed multinomial logistic regression analysis combined with random forest as a complementary algorithm for modelling using the R-package “randomForest” by Andy Liaw and Matthew Wiener [8] to compare expressions in the three groups of patients (S1 Appendix).

### QPCR analysis

Expression of selected genes in monocytes, and PBMCs, was validated with qPCR analysis on the whole cohort of 85 patients as described in S1 Appendix. QPCR analysis of genes in whole blood and PBMCs was performed as in monocytes. The refinement of models from qPCR data of all 85 patients was performed as described in S1 Appendix.

### Coronary atherosclerotic plaques

Then, we validated the expression in coronary atherosclerotic plaques. We performed blinded qPCR analysis of extracts from coronary atherosclerotic plaques of 7 patients (patients 1 and 2 and 4–8) and 5 (out of 6) control samples, collected at University of Padova, Italy, described in reference [9]. This study conforms to the principles outlined in the Declaration of Helsinki. The vascular samples were classified according American Heart Association recommendation [10]. Five were classified as type 7 and two as type 6. Controls were derived from the fragment of coronary samples not affected by plaque formation and with similar expression of several reference genes. We also measured cytokines, chemokines and growth factors in the plasma of these patients and controls.

### Peripheral vascular plaques

Gene expressions patterns in peripheral arterial samples were analyzed previously with Illumina HumanHT-12 v3 Expression BeadChip (Illumina, San Diego, CA) analyzing 47,000 transcripts of all known genes, gene candidates, and splice variants in the Tampere Vascular Study [11–13]. This study conforms to the principles outlined in the Declaration of Helsinki. In the present study, extracts from 29 carotid, 15 abdominal and 24 femoral atherosclerotic plaques and 24 non-atherosclerotic left internal thoracic artery control samples were analyzed in detail. The samples were taken from patients subjected to open vascular surgical procedures in the Division of Vascular Surgery and Heart Centre, Tampere University Hospital. The study has been approved by the Ethics Committee of Tampere Hospital District. Three plaques were classified as type 3, 4 as type 4, 20 as type 5 and 30 as type 6. The type of 11 plaques could not be determined. Even though the results from plaques were not replicated with qPCR, the accuracy of the RNA micro-array array has been verified by TaqMan qPCR for many other genes in samples from the Tampere Vascular Study [14].

### Other measurements

Total and HDL-cholesterol and triglyceride levels were determined with enzymatic methods (Boehringer Mannheim). LDL-cholesterol levels were calculated with the Friedewald formula. Plasma glucose was measured with the glucose oxidase method (on Vitros 750XRC, Johnson & Johnson). Hs-CRP (Beckman Coulter) was measured on an Image 800 Immunochemistry System; Hs-Troponin T (TNT) on a Modular E system (Roche Diagnostics). All laboratory assessments were performed without knowledge of clinical data. Diabetes mellitus was defined as fasting serum glucose levels >125 mg/dl or therapy with oral hypoglycaemic agents or insulin. Plasma was used to analyze cytokine, chemokines and growth factors using the Bioplex instrumentation (Bio-Rad). We used the human cytokine 27-plex panel according to the manufacturer's specificity [9]. Cytokines were analyzed with Assayfit Pro (AssayCloud, Netherlands) using a five parameters logistic regression for the interpolation of calibration points.

### Statistical analysis

Two groups of continuous variables were compared with Mann Whitney test; three groups with Kruskal–Wallis test followed by Dunn’s multiple comparisons test. Categorical data were compared with Fisher’s exact test. RNA expressions were compared with paired Friedman test followed by Dunn’s multiple comparisons test. Non-parametric correlations between expression in monocytes and atherosclerotic plaques or between expression in monocytes and blood parameters were determined (GraphPad Prism 6). ROC, Kaplan-Meier and COX proportional hazards regression analysis (MedCalc statistical software) was performed to determine the additive diagnostic value of selected genes in separating stable CAD patients with versus without new ischemic event to age, gender, (ex)-smoking, BMI, blood pressure, type-2 diabetes, HDL- and LDL-cholesterol, triglycerides and hs-C-reactive protein. P-values of less than 0.05 were considered as statistically significant. We calculated power based on sample size, group means and standard deviations and sample ratio (at 1% error rate).

## Results

### Patients

Baseline characteristics of patients with stable CAD with (n = 42) and without a new ischemic event (n = 24) and ACS (n = 19) are shown in Table 1. There was no baseline difference in stable CAD patients with versus without a new ischemic event. ACS patients had higher hs-CRP and LDL-cholesterol levels than the two groups of stable CAD patients. ACS patients were somewhat older than stable CAD patients who developed a new ischemic event and tended to be treated less often with a statin. Use of blood pressure lowering and antiplatelet drugs was not different. All ACS patients had received P2Y12 inhibitors and aspirin at time of hospitalization, before blood sampling.

**Table 1:** Demographic and clinical characteristics of stable CAD patients according to new coronary event and ACS patients.

	Stable CAD Without event (n = 42)	Stable CAD With event (n = 24)	ACS (n = 19)	P-value
Follow-up (days)	1943±369	2058±247 (NS)	--	--
Diseased vessels (0, 1, 2, 3; n)	0, 13, 16, 13	0, 8, 12, 4	2, 7, 7, 3	0.380
Age (years)	59±6.4	55±7.2	62±11^$^	0.016
Sex (n and % male)	36 (86)	22 (92)	15 (79)	0.492
SBP (mmHg)	142±19	142±20	140±21	0.890
DBP (mmHg)	80±12	80±14	83±13	0.651
Smoker; ex-smoker (n and %)	12;21 (29; 50)	7;11 (29; 46)	6;7 (31; 37)	0.897
BMI (kg/m^2^)	27±2.9	26±4.0	28±4.6	0.222
Type 2 diabetes (n and %)	6 (14)	4 (17)	0 (0)	0.188
TG (mg/dL)	149±92	111±38	147±85	0.149
LDL-C (mg/dL)	87±32	92±29	122±52**/^$^	0.0031
HDL-C (mg/dL)	44±12	46±12	46±12	0.895
Hs-CRP (mg/L)	3.09±2.66	2.28±2.28	8.56**/^$ $^	0.009
Hs-TNT (ng/μl) admission	--	--	0.27±0.43	--
Hs-TNT (ng/μl) peak	--	--	1.20±1.38	--
ACE-inhibitor use (n and %)	12 (29)	3 (13)	4 (21)	0.164
Angiotensin-II-receptor antagonist use (n and %)	6 (15)	4 (17)	1 (5.2)	0.317
Beta-blocker use (n and %)	21 (50)	11 (46)	7 (37)	0.634
Ca-antagonist use (n and %)	8 (19)	4 (17)	1 (5.2)	0.374
Statin use (n and %)	23 (55)	16 (67)	6 (32)	0.069
Antiplatelet drug (n and %)	29 (69)	19 (79)	9 (47)	0.082

Data shown are means ± SD. Abbreviations: BMI, body mass index; C, cholesterol; DBP, diastolic blood pressure; hs-CRP, high sensitivity C-reactive protein; hs-TNT: high sensitivity troponin T; SBP, systolic blood pressure; TG, triglycerides. Three groups of continuous variables were compared with Kruskal-Wallis followed by Dunn’s multiple comparison test; two groups were compared with Mann-Whitney test. Categorical data were compared with Fisher’s exact test.

** P<0.001 compared to stable CAD patients without event during follow-up

^$^ P<0.05 and

^$ $^ P<0.01 compared to stable CAD patients with event during follow-up. P-value for statin use comparing total group of stable CAD patients with ACS patients was 0.03.

### RNA sequencing and validation of gene expression by qPCR analysis

The full set of RNA sequencing data is available at https://www.ncbi.nlm.nih.gov/geo/query/acc.cgi?acc=GSE129935. Analysis of RNA sequencing data identified 20 genes with differential expression according to the patient group (S1 Appendix). We validated their expression with qPCR (Table 2). MT-COI, COX10 and STRN were different between stable CAD patients with and without new ischemic event. RNA expression of COX10 was similar in ACS patients and stable CAD patients with new ischemic event. STRN increased from stable CAD patients without new ischemic event to stable CAD patients with ischemic event to ACS patients. RNA expressions of SMIM9, TET2, RNF121, KDM5A, PRRC2C, TAF1, WNK1, ZNF484, AAMP and STK6 were different in ACS patients than in the two groups of stable CAD patients; their RNA expressions were similar in stable CAD patients with and without new ischemic event (Table 2).

**Table 2:** RNA expressions, measured with qPCR, in monocytes from stable CAD patients according to new ischemic event and ACS patients.

Gene	Stable CAD Without event (n = 42)	Stable CAD With event (n = 24)	ACS (n = 19)	P-value
MT-COI	1.08±0.31	0.89±0.25**	0.97±0.11	0.026
COX10	1.15±0.39	0.99±0.23*	0.94±0.13**	0.028
STRN	0.93±0.22	1.05±0.21*	1.28±0.21***^/^^$ $^	<0.0001
SMIM19	0.86±0.37	0.86±0.38	1.11±0.17*^/^^$^	0.027
TET2	0.84±0.39	0.86±0.30	1.12±0.19**^/^^$^	0.0083
RNF121	0.86±0.32	0.86±0.37	1.12±0.16**^/^^$^	0.0082
KDM5A	0.77±0.41	0.77±0.31	1.12±0.18***^/^^$ $^	0.0007
PRRC2C	0.77±0.39	0.87±0.35	1.34±0.37***^/^^$ $ $^	<0.0001
TAF1	0.75±0.35	0.77±0.37	1.26±0.28***^/^^$ $ $^	<0.0001
WNK1	0.81±0.31	0.87±0.28	1.35±0.23***^/^^$ $ $^	<0.0001
ZNF484	0.84±0.47	0.82±0.46	1.68±0.39***^/^^$ $ $^	<0.0001
AAMP	1.01±0.15	1.03±0.16	0.87±0.13***^/^^$ $^	<0.001
STK36	0.99±0.20	1.05±0.19	1.18±0.15***^/^^$ $^	<0.001

Data shown are means ± SD of ratios compared to healthy controls. Three groups of continuous variables were compared with Kruskal-Wallis followed by Dunn’s multiple comparison tests.

* P<0.05

** P<0.01

*** P<0.001 compared to stable CAD patients without event during follow-up

^$^ P<0.05

^$ $^ P<0.01

^$ $ $^ P<0.001 compared to stable CAD patients with event during follow-up. Abbreviations: AAMP: angio associated migratory cell protein; COX10: cytochrome oxidase 10; KDM5A: lysine demethylase 5A; MT-COI: mitochondrial cytochrome c oxidase, subunit I; PRRC2C: proline rich coiled-coil 2C; RNF121: ring finger protein 121; STK36: **s**erine threonine kinase 36; SMIM19: small integrated membrane protein 19; STRN: striatin; TAF1: TATA-box binding protein associated factor 1; TET2: tet methylcytosine dioxygense 2; WNK1: WNK lysine deficient protein kinase 1; ZNF484: zinc finger protein 484.

### MT-COI and STRN are associated with new ischemic event in stable CAD patients

**Fig 1:**
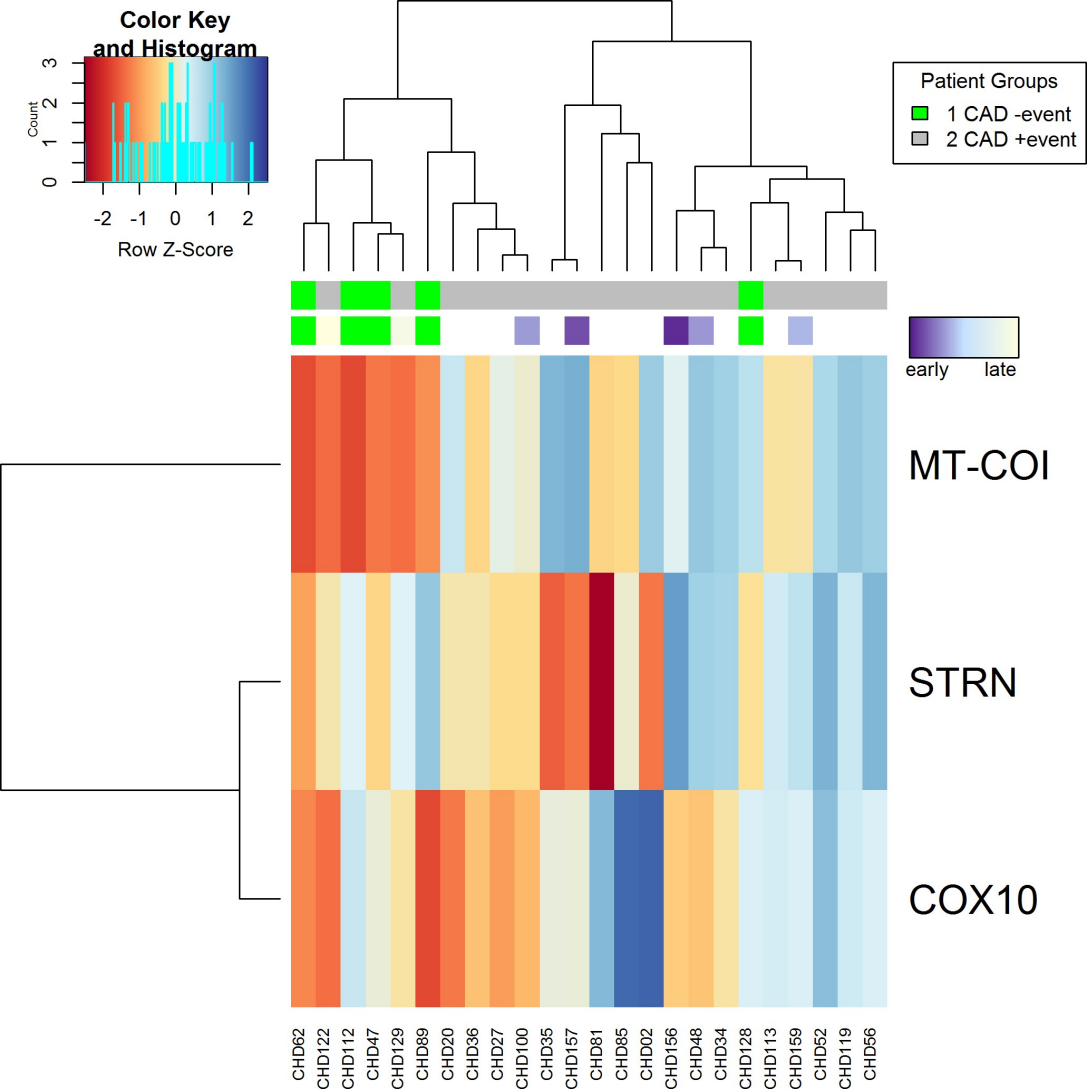
Gene expression heat map. Stable CAD patients with and without new ischemic event are separated according to gene expression of MT-COI, STRN and COX10 determined by RNA sequencing.

The gene expression heat map shows separation of stable CAD patients with and without new ischemic event according to the gene expression of MT-COI, STRN and COX10 determined by RNA sequencing (Fig 1).

**Fig 2:**
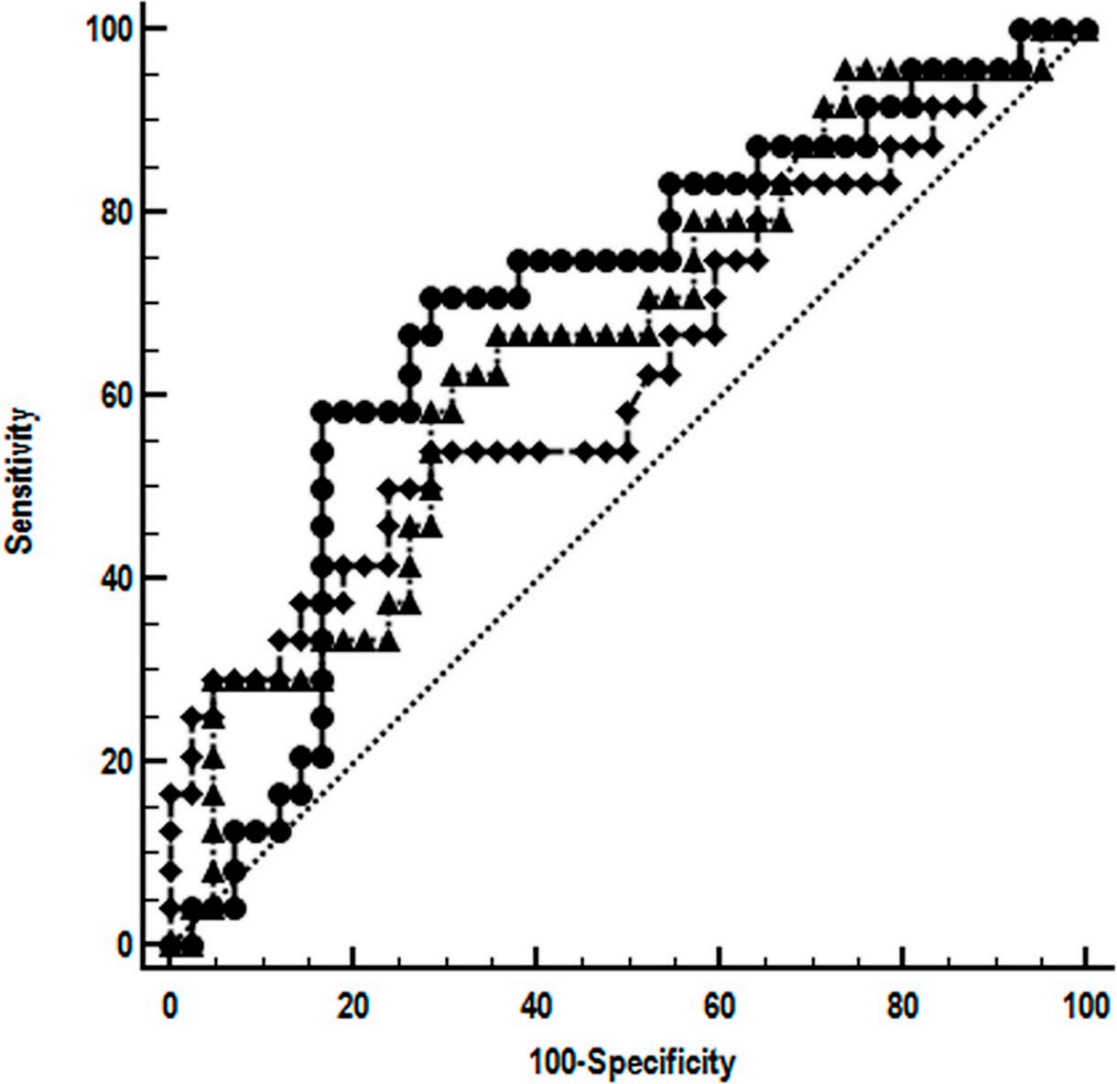
Receiver operating characteristics curve (ROC) analysis. RNA expressions of MT-COI, STRN and COX10 are related to occurrence of new ischemic event in stable CAD patients. Expressions were measured with qPCR. The area under curve was 0.69 (95% CI: 0.57–0.80) for MT-COI (circles), 0.66 (95% CI: 0.53–0.77) for STRN (triangles), and 0.63 (95% CI: 0.51–0.75) for COX10 (diamonds). The ROC curves were not statistically different.

ROC analysis confirmed that MT-COI, STRN and COX10 RNA are related to new ischemic event in CAD patients (Fig 2).

**Fig 3:**
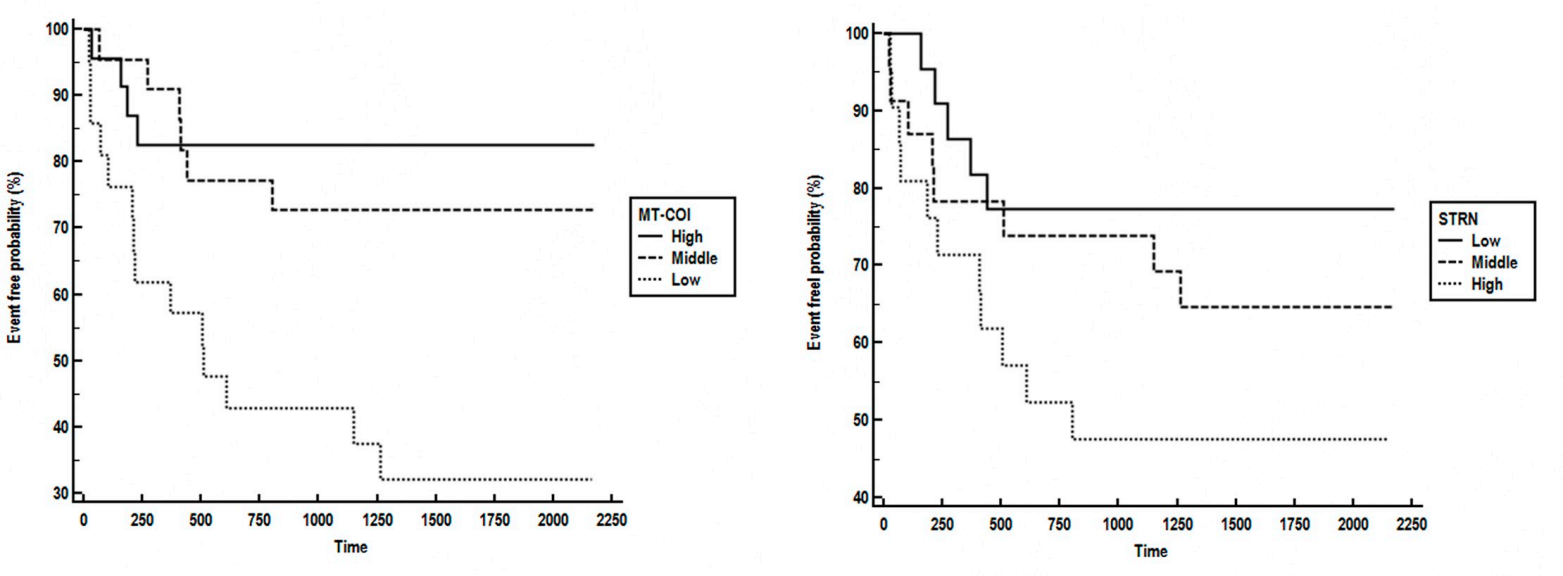
Kaplan-Meier analysis. Kaplan-Meier curves show that low RNA expression of MT-COI (left) and high RNA expression of STRN (right) were related with a shorter time between blood sampling and occurrence of new ischemic event. Adjusted HR, determined by COX proportional hazards regression analysis, of a new ischemic event in patients in lowest compared to patients in highest tertile of MT-COI was 5.14 (95% CI: 1.85–14). Adjusted HR of a new ischemic event in patients in highest compared to patients in lowest tertile of STRN was 2.88 (95% CI: 1.06–7.87). Adjustment was made for age, gender, (ex)-smoking, BMI, blood pressure, type-2 diabetes, HDL- and LDL-cholesterol, triglycerides and hs-C-reactive protein.

Power for separation of patients with and without new ischemic event was 0.97 for MT-COI (at 1% error rate), 0.90 for STRN and 0.77 for COX10. The accuracy for separating stable CAD patients with and without a new ischemic event increased from 67 to 80% by adding MT-COI and STRN to established cardiovascular risk factors age, gender, (ex)-smoking, BMI, blood pressure, type-2 diabetes, HDL- and LDL-cholesterol, triglycerides and hs-C-reactive protein. AUC increased from 0.66 (0.53–0.77) to 0.84 (0.73–0.92). Kaplan-Meier curves showed that MT-COI and STRN were related with the time between blood sampling and occurrence of new ischemic event (Fig 3).

COX proportional hazards regression analysis confirmed that MT-COI and STRN, but not COX10, were independently related to time to new event adjusting for age, gender, (ex)-smoking, BMI, blood pressure, type-2 diabetes, HDL- and LDL-cholesterol, triglycerides and hs-C-reactive protein. HR was 5.94 (95% CI: 2.47–14) for MT-COI and 4.47 (95% CI: 1.75–11) for STRN. That COX10 was not independently related may be due to the high correlation between MT-COI and COX10 (Rs = 0.45; P<0.0001).

### MT-COI, WNK1, STRN, COX10 and ZNF484 separate ACS from stable CAD patients

**Fig 4:**
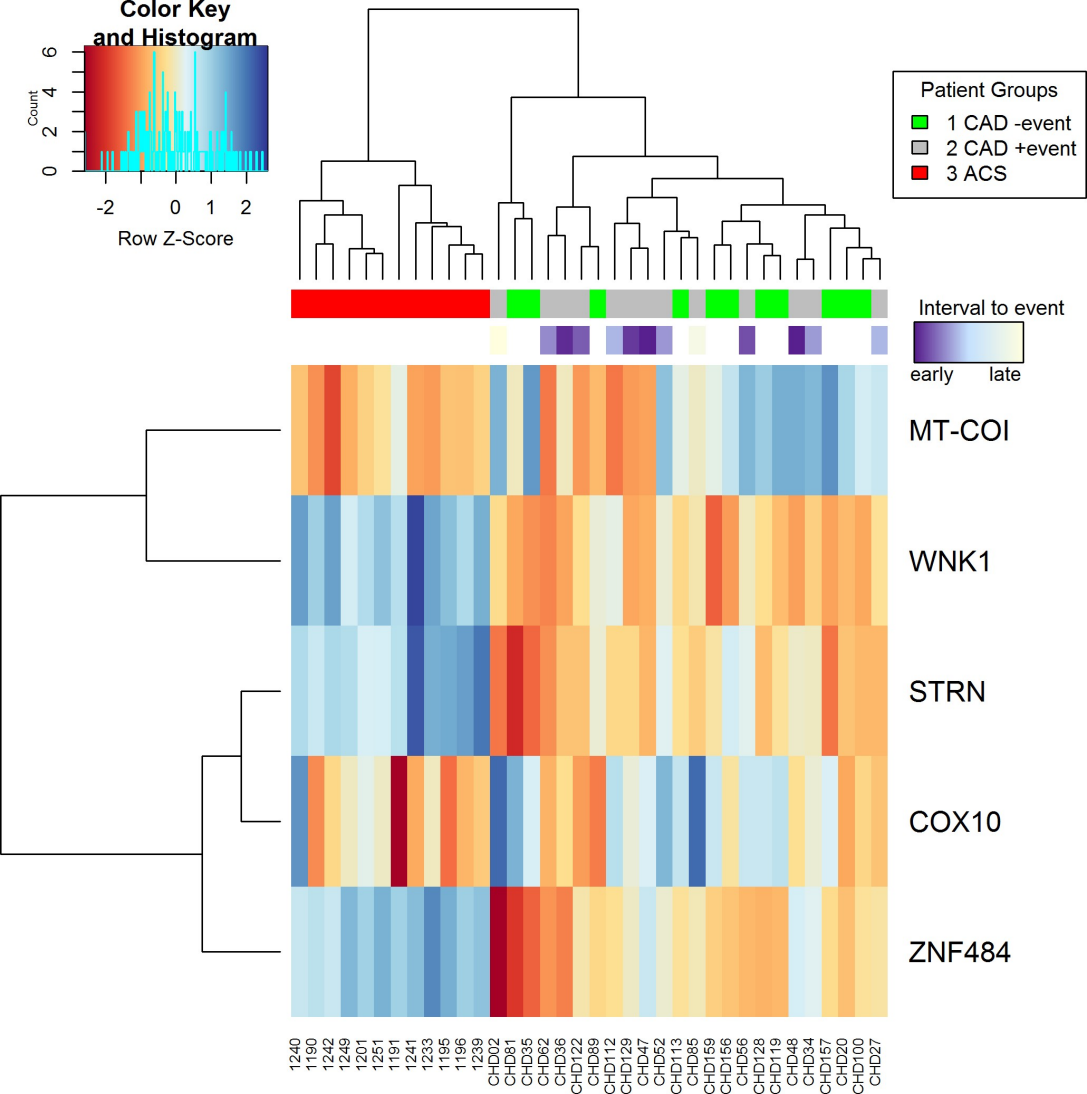
Gene expression heat map. ACS patients are separated from two groups of table CAD patients according to gene expression of MT-COI, WNK1, STRN, COX10, and ZNF484 determined by RNA sequencing.

The gene expression heat map shows complete separation of ACS from stable CAD patients with and without new ischemic event according to the gene expression of MT-COI, WNK1, STRN, COX10 and ZNF484 determined by RNA sequencing (Fig 4).

**Fig 5:**
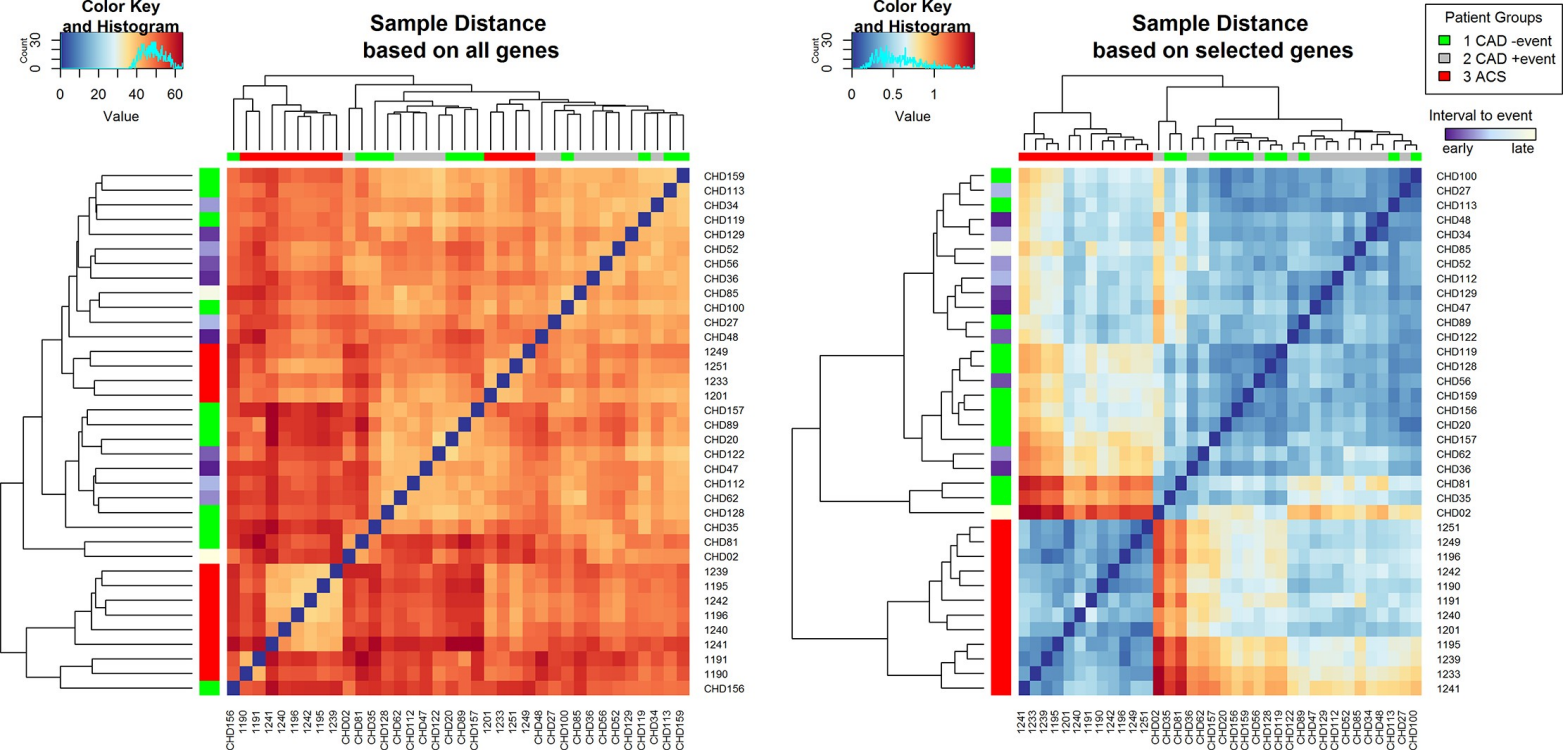
Clustering according to sample distance. Samples were clustered according to Euclidean distance of RNA sequencing expression profiles of all genes or profiles of genes selected by our models, i.e. MT-COI, WNK1, STRN, COX10, and ZNF484. The clustering by the selected genes shows smaller distances than those obtained by all genes. Orange / red refers to larger distance and blue to smaller. Furthermore, with the selected genes the ACS patients form a homogenous cluster.

Finally, these four genes significantly improved the clustering of patients into three groups compared to the whole collection of genes with significant differences in expression in the 3 groups in RNA-Seq analysis (Fig 5).

We then performed modelling in search of minimal gene groups which discriminated between ACS patients and the whole group of stable CAD patients, independent of a new ischemic event. We identified three models containing 3 genes which distinguished ACS from the whole group of CAD patients with an accuracy of 90%; COX10 and ZNF484 were present in all 3 models. The minimal model, with an accuracy of 90% consisted of COX10 and ZNF484. The sensitivity of COX10 and ZNF484 was 89% (95% CI: 52–99%). The specificity was 91% (95% CI: 52–99%). AUC was 0.96 (95% CI: 0.89–0.99) (Table 3).

**Table 3:** Models for separating ACS from stable CAD patients (stable CAD patients with and without new ischemic event combined).

Genes	Accuracy (%)
COX10, WNK1, ZNF484	91±7.0
COX10, PRRC2C, ZNF484	91±5.7
COX10, SMIM19, ZNF484	91±6.1
COX10, ZNF484	90±6.1

Data are means ± SD. Only models with an accuracy of ≥ 90% are presented. The sensitivity of COX10 and ZNF484 was 89% (95% CI: 52–99%). The specificity was 91% (95% CI: 52–99%).

We then determined if RNA expressions correlated with TNT peak levels in ACS patients. MT-COI (Rs = -0.045; p = 0.86), COX10 (Rs = -0.12; p = 0.61), STRN (Rs = -0.21; p = 0.39), WNK1 (Rs = -0.041; p = 0.87) and ZNF484 (Rs = -0.044; p = 0.86) did not correlate with TNT.

### Comparison of gene expression in monocytes with that in PBMCs and whole blood

Originally, we measured gene expression in monocytes because they are the precursors of macrophages. However, dependency on monocytes may hamper further validation, because their isolation is difficult to implement in clinical practice. Therefore, we also measured gene expression in whole blood and PBMCs of the same patients (n = 20). The expressions of MT-COI (0.71±0.10 vs. 0.88±0.11), COX10 (1.06±0.16 vs. 0.99±0.14), STRN (1.04±0.19 vs. 1.02±0.13) and WNK1 (0.95±0.23 vs. 1.14±0.15), but not ZNF484 (1.65±0.44 vs. 0.76±0.14), in PBMCs matched with these in monocytes. The expressions of COX10 (1.11±0.23), STRN (1.04±0.22) and WNK1 (0.8800780.26) in whole blood corresponded to these in monocytes; that of MTCOI did not.

### RNA expression in monocytes is independent of cholesterol lowering and antiplatelet treatment

Finally, we divided CAD patients according to medical therapies. The expression of three RNA tertiles of MT-COI (χ^2^ = 3.78; p = 0.15), COX10 (χ^2^ = 1.92; p = 0.38), STRN (χ^2^ = 1.37; p = 0.50), WNK1 (χ^2^ = 2.75; p = 0.25) and ZNF484 (χ^2^ = 0.014; p = 0.99) did not depend on statin use. The number of patients in the three tertiles of MT-COI (χ^2^ = 2.27; p = 0.32), COX10 (χ^2^ = 3.62; p = 0.16), STRN (χ^2^ = 0.78; p = 0.68), WNK1 (χ^2^ = 2.75; p = 0.25) and ZNF484 (χ^2^ = 0.068; p = 0.97) did also not depend on the treatment with antiplatelet drugs.

### Validation of expression of genes in human atherosclerotic plaques

Table 4 shows that the expression of the COX10 gene was decreased in human coronary atherosclerotic plaques. Blood levels of cytokines IL-17, IFN-γ, TNF-α, the macrophage inflammatory protein (MIP)-1α and MIP-1β, the chemotactic protein MCP-1 and the growth factor VEGF were elevated in cases with atherosclerosis versus controls. COX10 correlated inversely with all of them. In contrast, the eosinophil chemotactic protein eotaxin was not different and COX10 did not correlate with it.

**Table 4:** COX10 in human coronary atherosclerotic plaques correlates inversely with blood levels of cytokines, chemokines and vascular endothelial growth factor.

Factor	Cases (n = 7)	Controls (n = 5)	P-value	Correlation with COX10 (R_s_)	P-value
COX10	0.27±0.18	1.01±0.070	<0.0001	--	--
IL-17	33±20	10±1.70	0.024	-0.62	0.05
IFN-γ	260±71	83±6.13	0.0005	-0.61	0.04
TNF-α	35±23	8.04±4.15	0.021	-0.70	0.014
MIP-1α	12±2.18	7.50±0.51	0.0015	-0.74	0.0079
MIP-1β	52±11	23±3.45	0.0002	-0.78	0.0039
MCP-1	44±6.58	35±1.97	0.012	-0.61	0.038
VEGF	36±20	7.89±1.57	0.011	-0.76	0.0053
Eotaxin	112±4.59	100±24	0.228	0.21	0.509

The data are means ± SD. Blood levels are expressed in pg/ml. Continuous variables were compared with Mann-Whitney test. In addition, non-parametric Rs and P-values for correlations with COX10 are shown. Abbreviations: IFN: interferon; IL: interleukin; MIP: macrophage inflammatory protein; MCP-1; macrophage chemotactic protein or C-C motif chemokine ligand 2 (CCL2); TNF: tumour necrosis factor; VEGF: vascular endothelial growth factor.

The expression of ZNF484 was increased (6.15±1.80 vs. 1.01±0.13; P = 0.028) in human coronary atherosclerotic plaques; but it did not correlate with cytokines, chemokines or VEGF. Expression of WNK1 was not significantly different (0.82±0.38 vs. 1.00±0.077; P = 0.260). Expressions of MT-COI were very variable most likely due to very low expressions.

**Fig 6:**
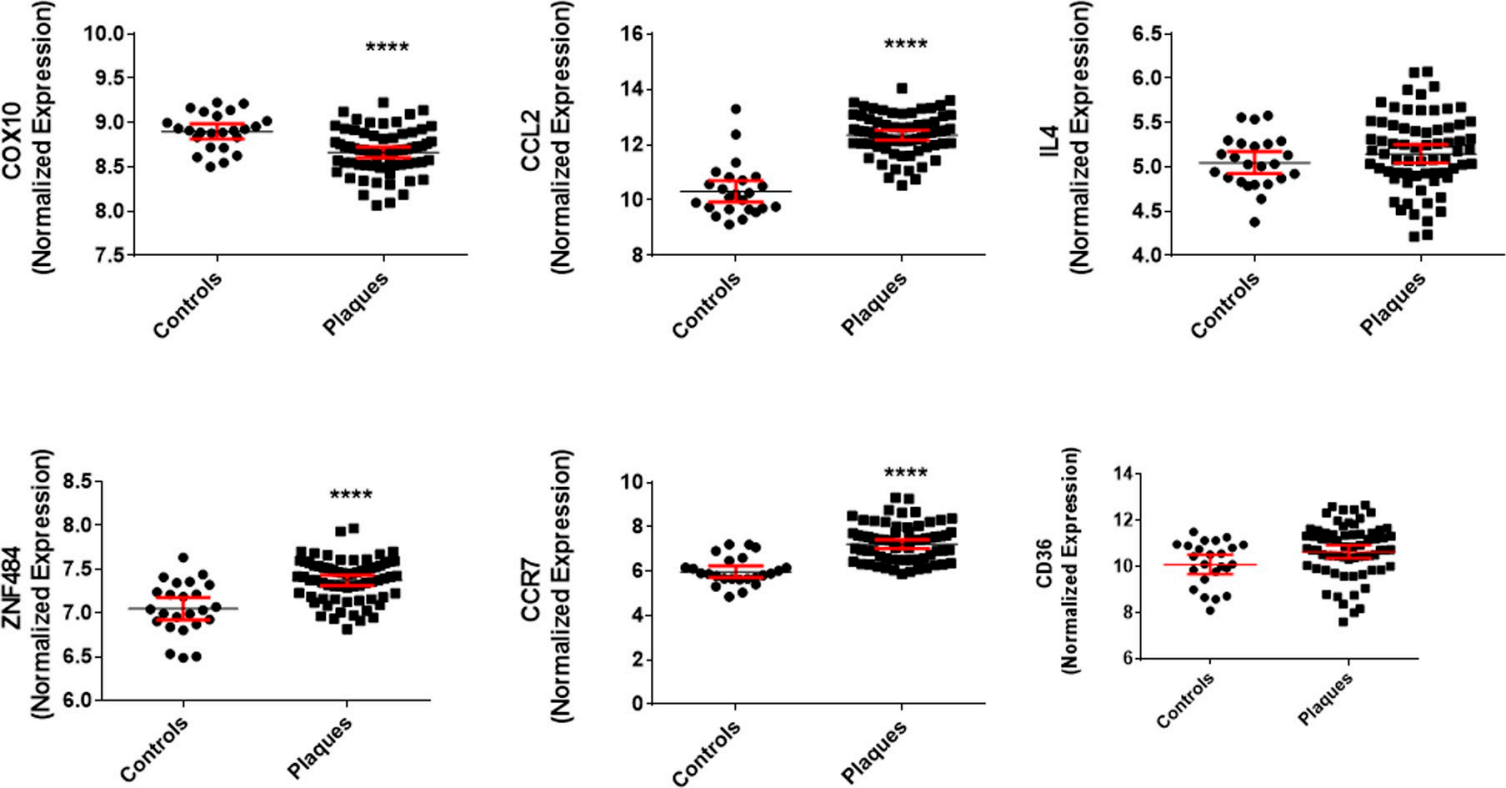
RNA expression of COX10 and ZNF484 and protein levels of markers of macrophage polarization. RNA expression of COX10 was lower and ZNF484 was higher in human atherosclerotic plaques than in control samples. Plaque levels of markers of M1 macrophages CCL2, CCR7 were higher. Plaque levels of markers of M2 macrophages CD36 and IL4 were not different between atherosclerotic plaques and control samples. The data are shown as scatter plots with geometric mean and 95% confidence intervals. **** P<0.0001 compared to controls.

As in coronary plaques, COX10 expressions were lower in peripheral atherosclerotic plaques than in control samples, whereas ZNF484 was higher. In addition, the expression of markers of inflammatory macrophages, CCL2 and CCR7 was higher in atherosclerotic plaques. In contrast, the expression of markers of M2 macrophages CD36 and IL-4 was not different between atherosclerotic plaques and control samples (Fig 6).

**Fig 7:**
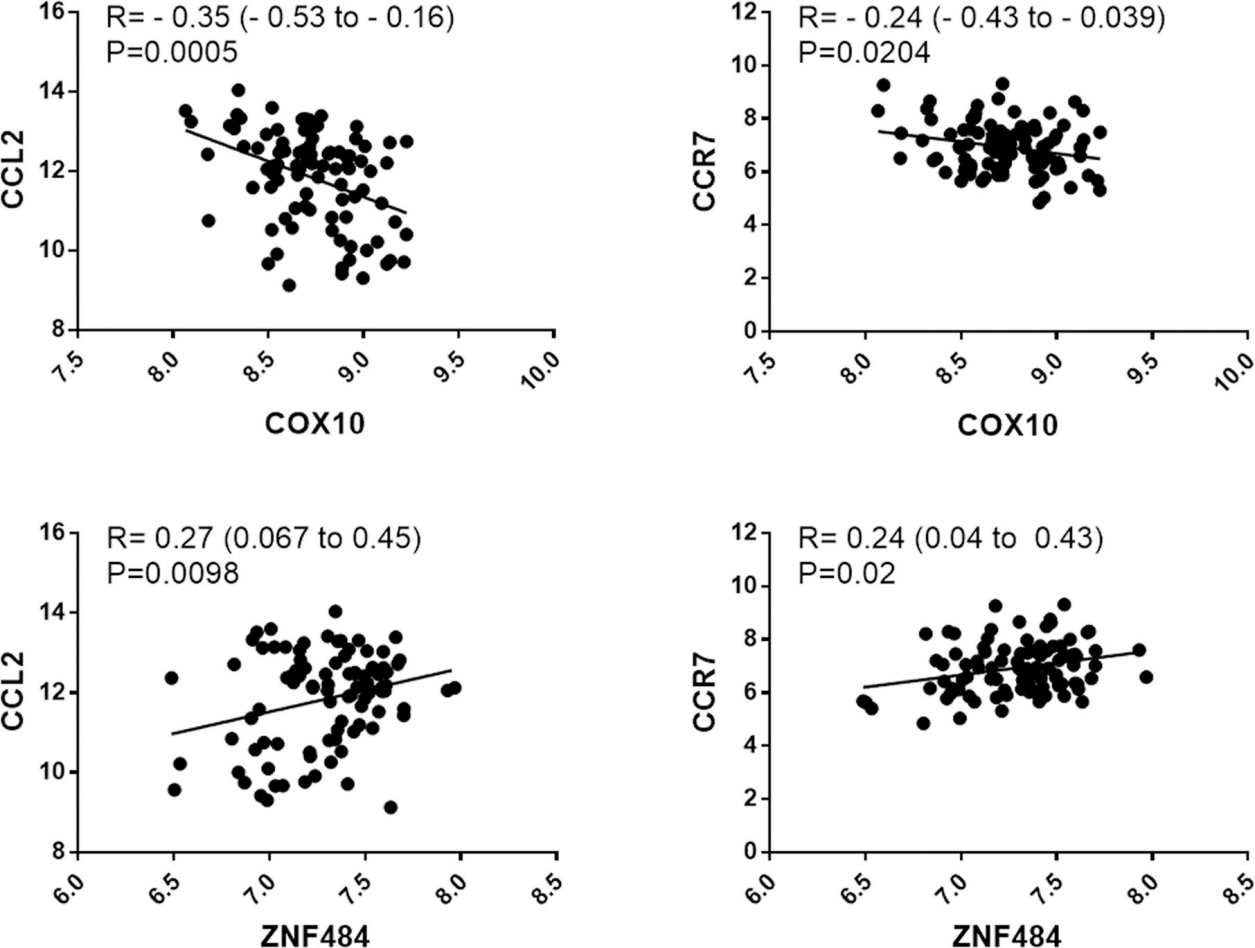
Correlation of COX10 and ZN484 with markers of protein levels of markers of macrophage polarization. COX10 and ZNF484 correlated with plaque levels of CCL2 and CCR7.

COX10 correlated negatively with CCL2 and CCR7; ZNF484 correlated positively with CCL2 and CCR7 (Fig 7). They did not correlate with markers of M2 macrophages.

## Discussion

RNA sequencing and qPCR analysis revealed that RNA expression levels of MT-COI and STRN improved separation of stable CAD patients according to their risk of a new ischemic event. RNA levels of COX10 were lower in CAD patients with a recurrent event, and resembled expression in patients with ACS and atherosclerotic plaques. ZNF484 was higher in ACS than in the two groups of stable CAD and also resembled expression in atherosclerotic plaques. Our data suggest that such RNA markers may allow us to intensify treatment upfront in patients with presumed progressive CAD, or to identify patients in which initiation of a new therapy is not directly needed because of very low risk. This hypothesis warrants to be tested in future studies.

The importance of a biomarker may also be underpinned by its role in the development of the disease. MT-COI is an important regulator of the cytochrome oxidase IV complex, the terminal node and rate limiting step in the mitochondrial electron transport chain, is associated with mitochondrial oxidative stress [15, 16], a condition associated with obesity, metabolic syndrome and type 2 diabetes [17, 18]. A single mutation in mouse MT-COI was associated with loss of COX activity [19], despite normal assembly of the complex IV [20]. Recently, we found that low MT-COI in isolated plaque macrophages in pigs was associated with complex coronary plaques and oxidized LDL [21].

COX10 is required for COX biogenesis [22] and is an important regulator of T cell activation, differentiation and signalling [23, 24]. For the first time we found that COX10 RNA expression was inversely related to blood levels of inflammation markers IL-17, IFN-γ, TNF-α, MIP-1α and MIP-1β, and MCP-1, and plaque protein levels of CCL2 and CCR7. CCL2 or MCP-1 is a marker of the persistent infiltration of low-grade inflammatory monocytes contributing to aggravated atherosclerosis [25]. In mice, we demonstrated that increased MCP-1 was associated with decreased interleukin-1 receptor-associated kinase-3 (Irak3 or IrakM), a regulator of innate immune function and increased mitochondrial reactive oxygen species production [26]. The motility CCR7 receptor may have a dual role in atherosclerosis. It leads to increased monocyte infiltration and macrophage accumulation in the growing atherosclerotic plaque [27]. But once plaques have been developed, it is required for plaque regression because it drives macrophage to egress from lesions [28].

The striatin protein family that is part of the striatin interaction phosphatases and kinases complex contains multidomain scaffolding proteins that play important role in cell adhesion [29]. Striatin-deficiency was found to increase vasoconstriction and decrease vascular relaxation [30]. It may decrease smooth muscle cell migration but enhance endothelial cell migration [31]. It assembles a membrane signalling complex necessary for rapid, nongenomic activation of endothelial NO synthase in endothelial cells [32].

For the first time we demonstrate an increase of ZNF484 in monocytes of unstable CAD patients as well as in human atherosclerotic lesions. ZNF484 is a Krüppel-associated box (KRAB) zinc-finger protein [33]. Overexpression of a group of KRAB-containing transcription factors in CD14+ monocytes of atherosclerotic patients resulted in increasing the expression of inflammation-related genes [34–37]. Our study shows that an increase of ZNF484 is associated with M1 macrophage polarization in human atherosclerotic plaques.

Interestingly, we did not observe differences in gene expression profiles according to treatment with statins or antiplatelet drugs. In light of the recent observation of the CANTOS trial [38, 39], it is hypothesized that there is a synergistic effect between the inflammatory burden leading to atherosclerosis on one side, and the traditional lipid burden. In line with these findings as well as ours, we may hypothesize that the measured RNA biomarkers in monocytes more reflect the inflammatory activation rather than the lipid metabolism. In addition, although activated platelets indeed do exert some pro-inflammatory effects, most if not all of the benefit of antiplatelet effects is clearly related to their antithrombotic properties. Although P2Y_12_ inhibitors have been shown to be able modulate inflammatory pathways in platelets and (for ticagrelor) neutrophils, the clinical benefit of these agents is still considered to be to most extent due to their ability to prevent arterial thrombosis.

A limitation of our study is the rather small number of patients, and the lack of validation in an independent population, thus requiring independent prospective validation. But it is noteworthy that differences in RNA expression patterns of COX10 and ZNF484 between atherosclerotic plaques and control arteries are similar as those between expression patterns in monocytes from ACS vs. stable CAD patients. Here we found that low MT-COI and STRN were related to a faster development of an event. Because of the low numbers, we were not able to categorize patients according to the type of event. We are aware that by including new biomarkers in clinical validation the volume of per-patient biomarker measurements for screening, monitoring, and diagnosing is poised to increase substantially. However, our research is timely because the number of applications utilizing real-time PCR (qPCR) is expanding rapidly. The improved accuracy of results relative to end-point PCR, multiplexing capability which decreased analysis cost, and reduced time are factors contributing to the growth of qPCR. For example, innovative technology allows to directly amplify a range of RNA targets from crude samples in-field in under 60 minutes via a closed tube real-time one step reaction without the need for a separate lab based nucleic acid extraction process. Especially the use of mcrochips will lead to rapid and sensitive detection of multiple targets possibly within 20 minutes [40, 41]. Portable microfluidic platforms may be very attractive in developing point-of-care diagnostics and precision medicine [42]. In additon, several machine-learning algorithms such as random forest, logistic regression, gradient boosting, and neural networks are now available to improve risk prediction [43–45]. In this study we used a non-invasive approach by performing analyses in peripheral blood because this is more convenient and translatable to clinical practice. On the other hand, a followup study should compare gene expression in both peripheral whole blood samples and coronary sinus blood samples in order to validate selected RNAs as cardiac markers.

## Conclusion

This work showed that MT-COI and STRN predict a new ischemic event in stable CAD patients whereas COX10 and ZNF484 have the potential to discriminate between stable CAD and ACS. Our data warrants further investigation of their interaction in atherosclerosis, plaque rupture and the development of unstable cardiovascular diseases.

## Supporting information

S1 Appendix (DOCX)
