# Supplementary material for: RNA-sequencing reveals that STRN, ZNF484 and WNK1 add to the value of mitochondrial MT-COI and COX10 as markers of unstable coronary artery disease
Source: PLoS One. 2019 Dec 10;14(12):e0225621. doi: 10.1371/journal.pone.0225621 (PMC6903720; doi:10.1371/journal.pone.0225621)
Supplement: S1 Appendix — (DOCX) [file pone.0225621.s001.docx]

PONE-D-19-13156R1

**RNA-sequencing reveals New Markers of Unstable Coronary Artery Disease**

**S1 Appendix**

**Extended materials and methods.**

**RNA sequencing**

RNA from monocytes was isolated at KU Leuven as described previously [1]. RNA concentration was determined using the Qubit 2.0 fluorometer (Thermo Fisher Scientific). Average RNA concentration was 122 ng/μl; ranging from 78-382 ng/μl. RNA quality was controlled using the Fragment Analyzer automated capillary electrophoresis system (Advanced Analytical Technologies). Average RQN value was 9.9, indicating very high RNA quality [2]. RNA sequencing (RNA-Seq) was performed by Biogazelle (Gent, Belgium). Libraries for mRNA sequencing were prepared using the TruSeq stranded mRNA sample prep kit (Illumina). In one run we analyzed samples from three groups of patients. Group 1: stable CAD patients without new ischemic (n=11); group 2: stable CAD patients with new event (n=13); group 3: ACS patients (n=12). Libraries for mRNA sequencing were prepared using the TruSeq stranded mRNA sample prep kit (Illumina). In brief, the starting material (100 ng) of total RNA was mRNA enriched using the oligodT bead system (Illumina). The isolated mRNA was subsequently fragmented using enzymatic fragmentation. Then first strand synthesis and second strand synthesis were performed, and the double stranded cDNA was purified (Agencourt AMPure XP, Beckman Coulter). The cDNA was end repaired, 3’ adenylated and Illumina sequencing adaptors ligated onto the fragments ends, and the library was purified (Agencourt AMPure XP). The polyA+ RNA stranded libraries were pre-amplified with PCR and purified (Agencourt AMPure XP). The libraries size distribution was validated, and quality inspected on the 2100 Bioanalyzer (high sensitivity DNA chip, Agilent). High quality libraries were quantified using the Qubit fluorometer (Life Technologies), the concentration was normalized, and the samples were pooled according to the project specification. Single-end sequencing (75 bp) was performed on NextSeq 500 instrument (Illumina) according to the manufacturer’s instructions (Illumina). Data analysis consisted of 3 main steps, each one with a quality check: data preparation, read mapping, and RNA quantification. At first, raw reads were evaluated using FastQC (v0.11.3). Adapter content was removed using Trimmomatic (v0.35; Bolger, Bioinformatics). Only reads with very low quality (Phred quality < 5) or very short reads (length < 25 nt) were trimmed before mapping using Trimmomatic as well. Top Hat (v2.1.0; Kim, Genome Biol) was used to align trimmed RNA-Seq reads to the reference genome and for the identification of splice junctions. Quality control after mapping was performed with QoRTs (v1.0.1; Hartley, BMC Bioinformatics). Finally, HTSeq (v0.6.1; Anders, Bioinformatics) was used to assemble the aligned sequences into genes, constructing a snapshot of the transcriptome. To guide the assembly process, an existing gene annotation was used. Annotation of the obtained sequences was performed using the Human Reference genome (GRCh38) and UCSC Genome Browser Reference transcriptome (Ensembl 84, LNCipedia 4.0). Raw counts were converted into FPKM values (fragments per kilobase per million read).

Spectral maps were generated using mpm R package based on DESeq2 normalized counts (DESeq2 R package; Love, Genome Biol; Biogazelle). Cumulative gene diversity was analyzed using QoRTs (v1.0.1; Hartley, BMC Bioinformatics) based on DESeq2 normalized counts. Raw read counts were normalized using the geometric mean based method implemented in DESeq2 R package. Transcripts with fewer than 6 read counts in all analyzed samples were excluded. This reduced the transcript annotations used in modelling from original 81,661 to 22,059 transcripts. A False Discovery Rate (FDR) of 5% was applied on p-values adjusted according to Benjamini & Hochberg method [3]. Outliers were managed according to DESeq2 algorithm [4].

We took two approaches for identifying candidate transcripts. First, we used Mann-Whitney test in search of transcripts separating stable CAD patients with and without a new coronary event. Then, in search of transcripts with the highest potential of separating the three group using a logistic multinomial regression model, in combination with random forest as a complementary algorithm for modelling using the R-package “randomForest” by Andy Liaw and Matthew Wiener [5].

The first approach led to the identification of another 7 genes: **angio associated migratory cell protein (AAMP); acylglycerol kinase (AGK); lysine demethylase 2A (KDM2A);** mitochondrial cytochrome c oxidase, subunit I (MT-COI); **neutrophil cytosolic factor 4 (NCF4); s**erine threonine kinase 36 (STK36) and striatin (STRN). The second approach led to the identification of 13 transcripts: lysine demethylase 5A (KDM5); proline rich coiled-coil 2C (PRRC2C); ring finger protein 121 (RNF121); RNA pseudouridine synthase D4 (RPUSD4); TATA-box binding protein associated factor 1 (TAF1); tet methylcytosine dioxygense 2 (TET2); THAP domain containing 11 (THAP11); tubulin folding cofactor C (TBCC); ubiquitin like 4A (UBL4A); WNK lysine deficient protein kinase 1 (WNK1) and zinc finger protein 484 (ZNF484). In addition, small integrated membrane protein 19 (SMIM19) was different in ACS patients compared to stable CAD patients with a new ischemic event, but not compared to stable CAD patients without event. Finally, cytochrome oxidase 10 (COX10) was different between ACS patients compared to stable CAD patients without a new event, but not compared to stale CAD patients with a new event.

**qPCR analysis**

Expression of selected genes was validated with quantitative real-time PCR (qPCR) analysis [1]. Previous to qPCR, RNA was stored at -80°C. As previously shown, a single freeze/thaw process had no significant effect on RNA integrity and quantity of plasma RNA [6]. First-strand cDNA was generated from total RNA with the SuperScript VILO cDNA synthesis kit (Invitrogen). qPCR was performed on a 7500 Fast Real-Time PCR system using Fast SYBRGreen master mix, according to the supplier protocols (Applied Biosystems). Oligonucleotides (Invitrogen) used as forward and reverse primers were designed using the “Primer Express” software (Applied Biosystems). Primers are presented in supplement table 1. RNA expression levels were calculated with the delta-delta-quantification cycle method (ΔΔC_q_) described by Livak and Schmittgen [7]. The C_q_ values for the gene of interest and the most stable housekeeping genes were determined for each sample to calculate ΔC_q,sample_ (C_q, gene of interest_ – mean C_q,housekeeping genes_), thus normalizing the data and correcting for differences in amount among RNA samples. In detail, HPRT1 (hypoxanthine phosphoribosyltransferase 1), SDHA (succinate dehydrogenase complex flavoprotein subunit A), TBP (TATA-box binding protein) and YWHAZ (tyrosine 3-monooxygenase/tryptophan 5-monooxygenase activation protein, zeta) were selected as most stable housekeeping genes using GeNorm [8]. The expression levels were related to lean calibrator individuals (n=7). Subsequently, ΔΔC_q_ (ΔC_q,sample_ – ΔC_q, control_) was determined, and the relative expression levels were calculated from 2^-ΔΔCq^. Replicate variability was within the set limit of 0.5 cycles for 98.2% of sample-target combinations in monocytes.

**Exhaustive modelling in search of minimal models for separation of three groups**

The refinement of models from the qPCR expression data of all 85 patients was performed with the R-packages caret (Kuhn 2008), nnet, and MASS (glm). The LASSO regularization previously employed for the RNA-Seq data not only deals with high dimensional data, it also works when collinearity is present. To avoid problems by collinearity we first calculated the Pearson correlation matrix for all qPCR measurements. We input this into the caret function *findCorrelation* with a threshold for absolute pair wise correlation of 0.8. First, univariate logistic regression models of ACS vs. all stable CAD patients were generated for each gene expression parameter. For each parameter tested we used the caret train function as a wrapper around a glm model. Although the glm function does not provide tuning parameters we set the caret train control to use 20 repeats of 5-fold cross-validations. This was done to get an averaged accuracy over the cross validation repeats. From the results of the final model fit of the train function we summarized the coefficient, the relative risk, the 95% confidence interval of the relative risk, the p-value of the coefficient, and the accuracy. From these results, the Kruskal-Wallis test of the expression values, and the suggested removal of highly correlated parameters a search for parameter combinations in multivariate logistic regression models was started. We used caret and glm in an R-script to evaluate all possible parameter combination down to models containing only two genes. Shrinking the models stepwise by manually removal of the least important parameter may be misleading since there is still considerable correlation of gene expression between the six genes. The parameter combinations were tested with the train function of the caret package each with 20 repeats of 5-fold cross validations. We recorded the average accuracy, the standard deviation of the accuracy, the AIC (Akaike information criterion), the residual deviance and calculated the p-value of the model against a null model from the likelihood ratio. The likelihood ratio test was also used to compare nested models. Then, we compared ACS with stable CAD patients with and without new events (three groups) as discussed above for comparison of two groups. The multinomial models employed the combination of the caret and nnet package instead of glm.

**S1 Table: Primers quantitative real-time PCR analysis.**

| **Gene Symbol** | **Forward primer** | **Reverse primer** | **R^2^** |
| --- | --- | --- | --- |
| AAMP | CTCTTGAAAGTGTGGCAGGT | GGTCCCAAATCCTGATGGTC | 0.995 |
| AGK | CAAGTGAAGAAGGCCACTGT | CCAGGAGTTTCTTGGCTTGT | 0.993 |
| KDM2A | TCAGAAAGAGTCCCTCAGCA | CACACTTGTCCATTGTGGGA | 0.999 |
| KDM5A | TCCTGAGCGGCTTGTATGTC | AACACGACTGACCCAAGTGT | 0,989 |
| **NCF4** | GAAGATCTCAGCAGCACTCC | ATGGCATCGTGTTGTAGACC | 0.998 |
| **PRRC2C** | CTGTAATAAAGAGGAGGAACCCG | CCACTGGTGTGGCTTCCTTT | 0,983 |
| **RNF121** | GTCCTCATCCTCATCGCAAC | CACTAGGAACCTCCACCAGT | 0,941 |
| RPUSD4 | CGAGCTACCGCATGGACGA | GACAAGTGAACTCGAAGCTGATG | 0.982 |
| **SMIM19** | GTGCCACCTACAGAGGAAACTT | CTGTCAGCTTGGTTTTGTGGC | 0,976 |
| **STK36** | TTGGGAGAGGAGCTGTTACA | AATCACATGCTATGGGCTGG | 0.993 |
| **STRN** | AACTTCCTTCATTGCAGCCA | CCAGTTCACTTTCAAGGGCT | 0.999 |
| **TAF1** | **GCCCCTTTGCCACTCAGATT** | **TGGAGGAGGCATCAACTTGC** | 0,963 |
| **TBCC** | AGCTCCGCATACACAGTACG | TCCTTGTCGATCTCCGGGTA | 0.984 |
| **TET2** | CCAGGAAAGAGGAGGAACCCG | CCACTGGTGTGGCTTCCTTT | 0.936 |
| **THAP11** | AACTGCGTGAGAAGGATCGG | GTACTGTCAACCAGTGCCCA | 0.937 |
| UBL4A | CAGCTACAGAGGGATTACGAGA | CACACTTAGTGCGACATGCAG | 0.961 |
| **WNK1** | ACAGATACAGTTGCTGCCCTAC | CCTTCTGTCATTTGGGCACG | 0,967 |
| **ZNF484** | TGGTGAGATCCCCAGTCAGA | TGTGTGTTCATCGTCTTTCCAC | 0,973 |
| **MT-COI** | CCACGGAAGCAATATGAAATGAT | CCTACGGTGAAAAGAAAGATGAATC | 0.837 |
| **COX10** | GGACTCCCCTCACAAGTTCTTACAT | GTGACATACATGCGTTTGAGGAA | 0.982 |

Reference List

1. Holvoet P, Vanhaverbeke M, Bloch K, Baatsen P, Sinnaeve P, Janssens S. Low MT-CO1 in Monocytes and Microvesicles Is Associated With Outcome in Patients With Coronary Artery Disease. J Am Heart Assoc. 2016;5(12). Epub 2016/12/07. doi: 10.1161/JAHA.116.004207. PubMed PMID: 27919931; PubMed Central PMCID: PMCPMC5210432.

2. Vermeulen J, De Preter K, Lefever S, Nuytens J, De Vloed F, Derveaux S, et al. Measurable impact of RNA quality on gene expression results from quantitative PCR. Nucleic Acids Res. 2011;39(9):e63. Epub 2011/02/15. doi: 10.1093/nar/gkr065. PubMed PMID: 21317187; PubMed Central PMCID: PMCPMC3089491.

3. Ghosh D. Incorporating the empirical null hypothesis into the Benjamini-Hochberg procedure. Stat Appl Genet Mol Biol. 2012;11(4). Epub 2012/08/02. doi: 10.1515/1544-6115.1735. PubMed PMID: 22850065.

4. Bourgon R, Gentleman R, Huber W. Independent filtering increases detection power for high-throughput experiments. Proc Natl Acad Sci U S A. 2010;107(21):9546-51. Epub 2010/05/13. doi: 10.1073/pnas.0914005107. PubMed PMID: 20460310; PubMed Central PMCID: PMCPMC2906865.

5. Svetnik V, Liaw A, Tong C, Culberson JC, Sheridan RP, Feuston BP. Random forest: a classification and regression tool for compound classification and QSAR modeling. J Chem Inf Comput Sci. 2003;43(6):1947-58. Epub 2003/11/25. doi: 10.1021/ci034160g. PubMed PMID: 14632445.

6. Cerkovnik P, Perhavec A, Zgajnar J, Novakovic S. Optimization of an RNA isolation procedure from plasma samples. Int J Mol Med. 2007;20(3):293-300. Epub 2007/08/03. PubMed PMID: 17671732.

7. Livak KJ, Schmittgen TD. Analysis of relative gene expression data using real-time quantitative PCR and the 2(-Delta Delta C(T)) Method. Methods. 2001;25(4):402-8. Epub 2002/02/16. doi: 10.1006/meth.2001.1262. PubMed PMID: 11846609.

8. Vandesompele J, De Preter K, Pattyn F, Poppe B, Van Roy N, De Paepe A, et al. Accurate normalization of real-time quantitative RT-PCR data by geometric averaging of multiple internal control genes. Genome Biol. 2002;3(7):RESEARCH0034. Epub 2002/08/20. PubMed PMID: 12184808; PubMed Central PMCID: PMCPMC126239.
